# Spatio-temporal pattern and associate factors of intestinal infectious diseases in Zhejiang Province, China, 2008–2021: a Bayesian modeling study

**DOI:** 10.1186/s12889-023-16552-4

**Published:** 2023-08-29

**Authors:** Zhixin Zhu, Yan Feng, Lanfang Gu, Xifei Guan, Nawen Liu, Xiaoxia Zhu, Hua Gu, Jian Cai, Xiuyang Li

**Affiliations:** 1 Department of Big Data in Health Science, and Center for Clinical Big Data and Statistics, The Second Affiliated Hospital, College of Medicine, Zhejiang University, Hangzhou, 310058 China; 2 Department of Infectious Disease Control and Prevention, Zhejiang Provincial Center for Disease Control and Prevention, Hangzhou, 310051 China; 3 The Cancer Hospital of the University of Chinese Academy of Sciences (Zhejiang Cancer Hospital), Institute of Basic Medicine and Cancer, Chinese Academy of Sciences, Hangzhou, 310022 China

**Keywords:** Intestinal infectious diseases, Spatial clustering, Climate factors, Socioeconomic condition, Hierarchical Bayesian model

## Abstract

**Background:**

Despite significant progress in sanitation status and public health awareness, intestinal infectious diseases (IID) have caused a serious disease burden in China. Little was known about the spatio-temporal pattern of IID at the county level in Zhejiang. Therefore, a spatio-temporal modelling study to identify high-risk regions of IID incidence and potential risk factors was conducted.

**Methods:**

Reported cases of notifiable IID from 2008 to 2021 were obtained from the China Information System for Disease Control and Prevention. Moran’s I index and the local indicators of spatial association (LISA) were calculated using Geoda software to identify the spatial autocorrelation and high-risk areas of IID incidence. Bayesian hierarchical model was used to explore socioeconomic and climate factors affecting IID incidence inequities from spatial and temporal perspectives.

**Results:**

From 2008 to 2021, a total of 101 cholera, 55,298 bacterial dysentery, 131 amoebic dysentery, 5297 typhoid, 2102 paratyphoid, 27,947 HEV, 1,695,925 hand, foot and mouth disease (HFMD), and 1,505,797 other infectious diarrhea (OID) cases were reported in Zhejiang Province. The hot spots for bacterial dysentery, OID, and HEV incidence were found mainly in Hangzhou, while high-high cluster regions for incidence of enteric fever and HFMD were mainly located in Ningbo. The Bayesian model showed that Areas with a high proportion of males had a lower risk of BD and enteric fever. People under the age of 18 may have a higher risk of IID. High urbanization rate was a protective factor against HFMD (RR = 0.91, 95% CI: 0.88, 0.94), but was a risk factor for HEV (RR = 1.06, 95% CI: 1.01–1.10). BD risk (RR = 1.14, 95% CI: 1.10–1.18) and enteric fever risk (RR = 1.18, 95% CI:1.10–1.27) seemed higher in areas with high GDP per capita. The greater the population density, the higher the risk of BD (RR = 1.29, 95% CI: 1.23–1.36), enteric fever (RR = 1.12, 95% CI: 1.00–1.25), and HEV (RR = 1.15, 95% CI: 1.09–1.21). Among climate variables, higher temperature was associated with a higher risk of BD (RR = 1.32, 95% CI: 1.23–1.41), enteric fever (RR = 1.41, 95% CI: 1.33–1.50), and HFMD (RR = 1.22, 95% CI: 1.08–1.38), and with lower risk of HEV (RR = 0.83, 95% CI: 0.78–0.89). Precipitation was positively correlated with enteric fever (RR = 1.04, 95% CI: 1.00–1.08), HFMD (RR = 1.03, 95% CI: 1.00–1.06), and HEV (RR = 1.05, 95% CI: 1.03–1.08). Higher HFMD risk was also associated with increasing relative humidity (RR = 1.20, 95% CI: 1.16–1.24) and lower wind velocity (RR = 0.88, 95% CI: 0.84–0.92).

**Conclusions:**

There was significant spatial clustering of IID incidence in Zhejiang Province from 2008 to 2021. Spatio-temporal patterns of IID risk could be largely explained by socioeconomic and meteorological factors. Preventive measures and enhanced monitoring should be taken in some high-risk counties in Hangzhou city and Ningbo city.

**Supplementary Information:**

The online version contains supplementary material available at 10.1186/s12889-023-16552-4.

## Introduction

Intestinal infectious disease (IID) remains one of the top listing causes of disease burden worldwide [1–4]. According to the World Health Organization (WHO), diarrhoeal disease was the second leading cause of death in children under five years old and was responsible for the deaths of 370 000 children in 2019 [3].

China was one of the 15 countries with high diarrhea burden [5]. In China, notifiable IID mainly incorporate cholera, poliomyelitis, bacterial dysentery (BD), amoebic dysentery (AD), typhoid, paratyphoid, hepatitis A/E, hand, foot and mouth disease (HFMD), and other infectious diarrhea (OID) defined as infectious diarrhea other than cholera, dysentery, typhoid/paratyphoid fever [6]. Owing to progress in sanitation status and public health awareness, the incidence of some typical and severe IID such as cholera, BD, AD, typhoid, and paratyphoid, decreased in the past decades, but the incidence of HFMD, hepatitis E, and OID were on the increase [7, 8]. HFMD, a neglected enteric infectious disease, was estimated to cause a huge burden in East and Southeast Asia, especially in China [9].

IID was mainly transmitted by the fecal–oral route upon ingestion of contaminated water or food [10]. Recent studies also revealed that regional heterogeneity in the occurrence of IID depends on economic development, local climate, and geographical conditions [11, 12]. Climate change and increasing extreme weather events accelerated the transmission of infectious diseases by impacting pathogen growth, survival of vectors and human behavior [13]. A better understanding of the epidemiology, seasonality, and spatio-temporal patterns of IID would be valuable for planning and adopting targeted preventive measures. Some previous studies had explored the spatio-temporal patterns and risk factors for the onset of IID but were subject to limitations such as short surveillance duration, less investigated factors, and single disease studied [14–18]. Zhejiang is a densely populated area situated in southeastern coastal China with a high incidence of IID [14, 15, 19, 20], and the spatio-temporal patterns of IID have yet to be identified. Thus, based on long-term continuous surveillance data of notifiable IID in Zhejiang Province from 2008 to 2021, the present study conducted a comprehensive investigation to give a full picture of the epidemiology of each IID and identify risk factors accounting for socioeconomic and climate aspects under spatio-temporal scales.

## Methods

### Study area and data sources

**Fig. 1:**
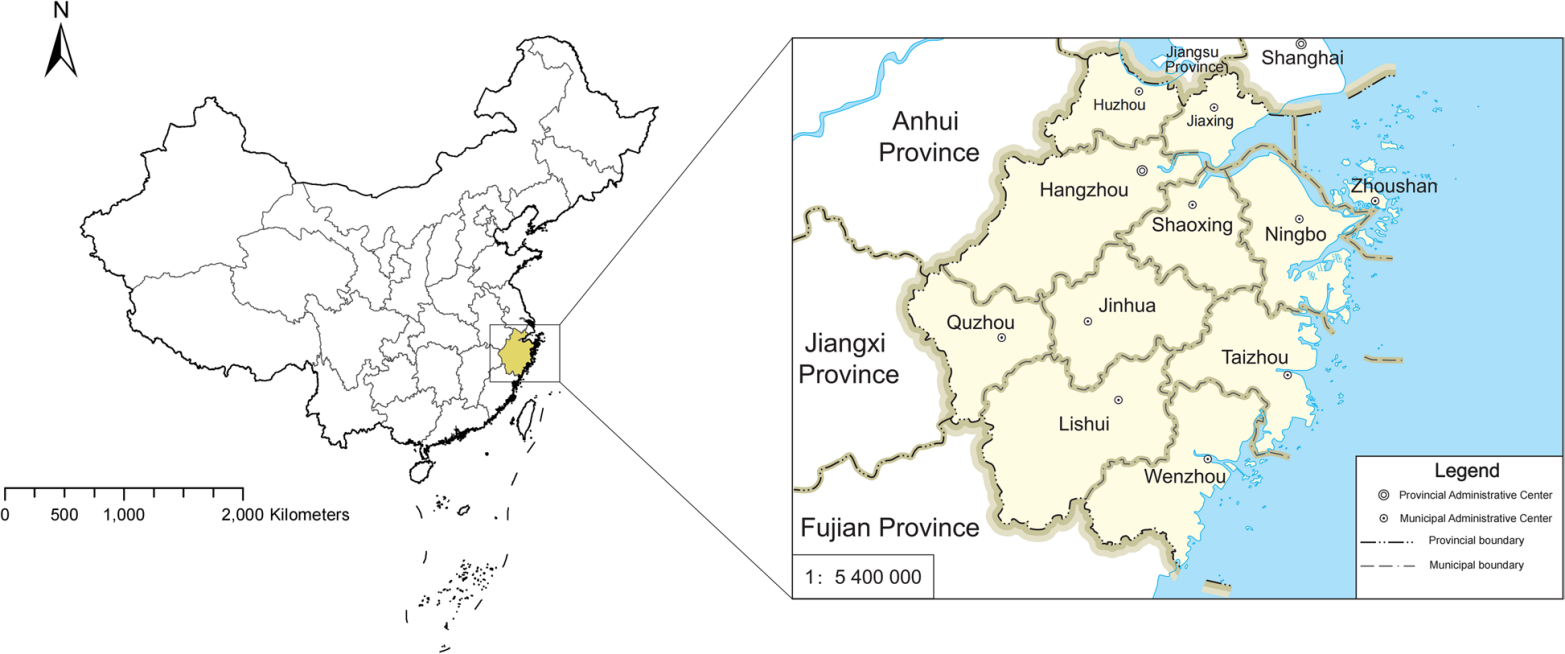
Geographic Location and administrative map of Zhejiang Province

Zhejiang is situated in southeastern coastal China between longitudes 118°E-123°E and latitudes 27°N–32°N, with a land area of 101 800 square kilometers. Zhejiang Province is administratively divided into 11 cities, which are further broken down into 92 counties (Fig. 1).

Reported cases of notifiable IID during 2008‒2021 were collected from the China Information System for Disease Control and Prevention, including cholera, BD, AD, typhoid, paratyphoid, hepatitis E, HFMD, and OID. County-level socioeconomic data including total population, proportion of male population, proportion of population under 18 years old, gross domestic product (GDP) per capita, urbanization rate, population density, and aquatic product output were obtained from the National Bureau of Statistics of China. Monthly meteorological data (surface pressure, temperature, precipitation, relative humidity, wind velocity, and sunlight duration) were obtained from the European Center For Medium-range Weather Forecasts [21]. The horizontal resolution of the meteorological data was 0.25° × 0.25° for latitude and longitude.

### Statistical analysis method

Typhoid and paratyphoid fevers were classified as enteric fever for subsequent analysis [22]. Choropleth maps of the incidence of each IID were produced using ArcGIS version 10.2 with shapefiles. For each IID, sequence diagram and radar chart with monthly incidence were used to display the long-term trend and seasonality.

### Spatial autocorrelation analysis

Global spatial autocorrelation analyses and the local indicators of spatial association (LISA) were used to explore the spatial patterns and the potential hotspots associated with the IID incidence at the county level. Global Moran’s I Index was applied to analyze global spatial autocorrelation. A positive value of Moran’s I Index implied a spatial clustering distribution while a negative value meant a spatial dispersing distribution, and a zero value implied a random spatial distribution. LISA was applied to identify significant hot spots (High-High), cold spots (Low-Low), and spatial outliers (High–Low and Low–High) by calculating local Moran’s I between a certain location and the average values of the surrounding regions. The spatial autocorrelation analyses were conducted using GeoDa software version 1.20.36.

### Bayesian hierarchical spatio–temporal model

In a Bayesian framework, monthly cases of BD, enteric fever, HFMD, and HEV were modelled respectively using hierarchical spatio–temporal regression. Socioeconomic variables (proportion of male population, proportion of population under 18 years old, GDP, urbanization rate, population density, and aquatic product output per capita) and meteorology variables (temperature, surface pressure, precipitation, relative humidity, wind velocity, and sunlight duration) were investigated as independent variables. The variance inflation factor diagnostic tool was used to test the collinearity of the selected covariates [23]. We standardized all the variables using z-score normalization to avoid numerical problems during the coefficient estimation procedure [24]. Infectious events are often not independent of each other, leading to overdispersion in infectious case data. The negative binomial regression model is more reliable than the Poisson regression model for explaining the overdispersion structure of the data [16, 25, 26]. Therefore, the observed number of HFMD cases in each county was assumed to follow a negative binomial (NB) distribution. BD, enteric fever, and HEV cases were assumed to follow zero-inflated negative binomial (ZINB) distributions respectively due to the large number of cells containing zero case counts in the dataset:

1 $$P\left({Y}_{it}={y}_{it}\right)=\left\{\begin{array}{c}{P}_{it}+\left(1-{p}_{it}\right){f\left(0\right)}_{ ,}{ y}_{it}=0\\ \left(1-{p}_{it}\right)f{\left({y}_{it}\right) }_{, }{ y}_{it}>0\end{array}\right.$$

2 $$f\left({y}_{it}\right)=\frac{\Gamma \left({y}_{it}+{\alpha }^{-1}\right)}{{y}_{it}!\Gamma \left({\alpha }^{-1}\right)}{\left(\frac{{\alpha }^{-1}}{{\alpha }^{-1}+{u}_{it}}\right)}^{{\alpha }^{-1}}{\left(\begin{array}{c}{u}_{it}\\ \overline{{\alpha }^{-1}+{u}_{it}}\end{array}\right)}^{{y}_{it}}$$

where ${\alpha }^{-1}$ is a dispersion parameter and *P* indicates the probability of being an excess zero. When *P* equal to 0, the model is equivalent to a negative binomial distribution. And

3 $${Y}_{it}\sim NB\left({u}_{it}\right)$$

4 $$\mathit{log}\left({u}_{it}\right)=\mathit{log}\left({E}_{it}\right)+{\theta }_{it}$$

5 $${\theta }_{it}={\alpha }_{0}+\sum\nolimits_{k}^{m}{\beta }_{k}{X}_{k}+{u}_{i}+{v}_{i}+{\varphi }_{t}+{\psi }_{it}$$

where ${Y}_{ij}$ is the number of intestinal infection cases in $i$ =1, 2,…,92 counties and time $t$; ${u}_{it}$ is the mean number of cases; ${E}_{it}$ is the expected number of cases (acting as an offset to control for population size); ${\theta }_{it}$ is the log relative risk of the IID; ${X}_{k}$ is the $k$-th variable included in the model; ${\alpha }_{0}$ quantifies the intercept fixed effect; ${\beta }_{k}$ quantify the fixed effects of the investigated variables.

${u}_{i}$ is a spatially structured random effect with mean zero and variance ${\sigma }_{u}^{2}$ to account for spatial autocorrelation, and ${v}_{i}$ is a spatially unstructured random effect with mean zero and variance${\sigma }_{v}^{2}$. These spatial components were modelled together using a Besag-York Mollie model [27]. The spatially structured random effect,${u}_{i}$, was modelled using conditional autoregressive priors [28], and the spatially unstructured random effect,${v}_{i}$, was modelled with an independent and identically distributed normal distribution. ${\varphi }_{t}$ is a temporally structured random effect, modelled with a first-order random walk latent model. ${\psi }_{it}$ is a spatiotemporal interaction effect to account for any residual spatiotemporal variation which was not captured by the spatial or temporal main effects and was assumed to be independently and identically distributed. Three types of models were modelled for each IID. Model I contained socioeconomic and climate variables and spatial terms (${u}_{i}+{v}_{i}$). Model II added a temporal term (${\varphi }_{t}$). Model III further contained a spatiotemporal interaction term (${\psi }_{it}$). The deviance information criterion (DIC) was used to assess and compare the fitness of the models and the beat model was selected [29]. The Bayesian models presented in this study were implemented using integrated nested Laplace approximation with the R-INLA package in the R programming language, version 4.2.2 [30]. 95% confidence intervals (CI) were used to represent the 2.5th to 97.5th percentiles of the posterior distribution of estimated IID incidence, and a *P* ≤ 0.05 was considered statistically significant. All tests were two-sided.

## Results

### Summary

**Fig. 2:**
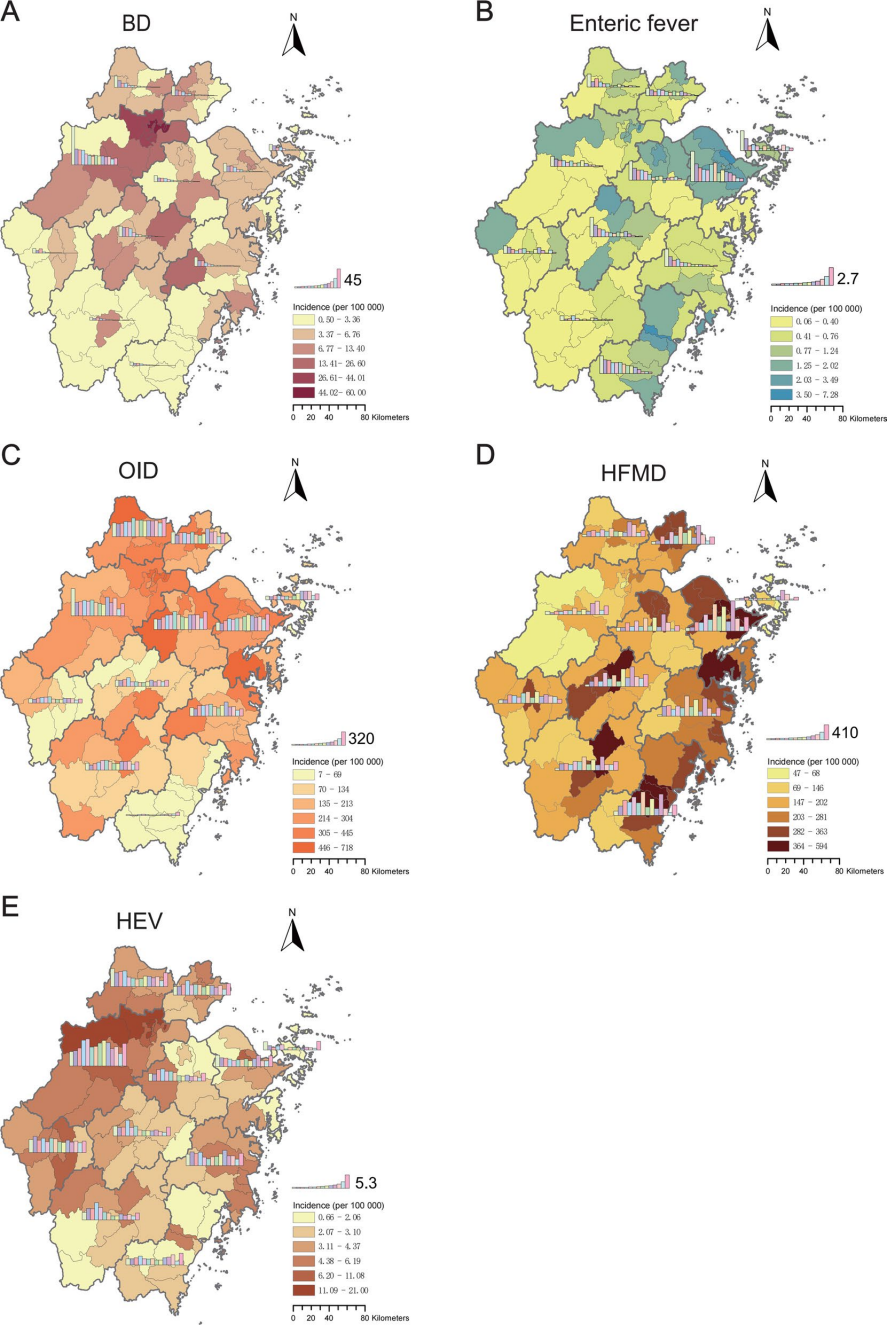
Spatial and temporal distribution of intestinal infectious disease incidence in Zhejiang Province during 2008‒2021. The city-level yearly incidence from 2008 to 2021 was reflected by histograms

**Fig. 3:**
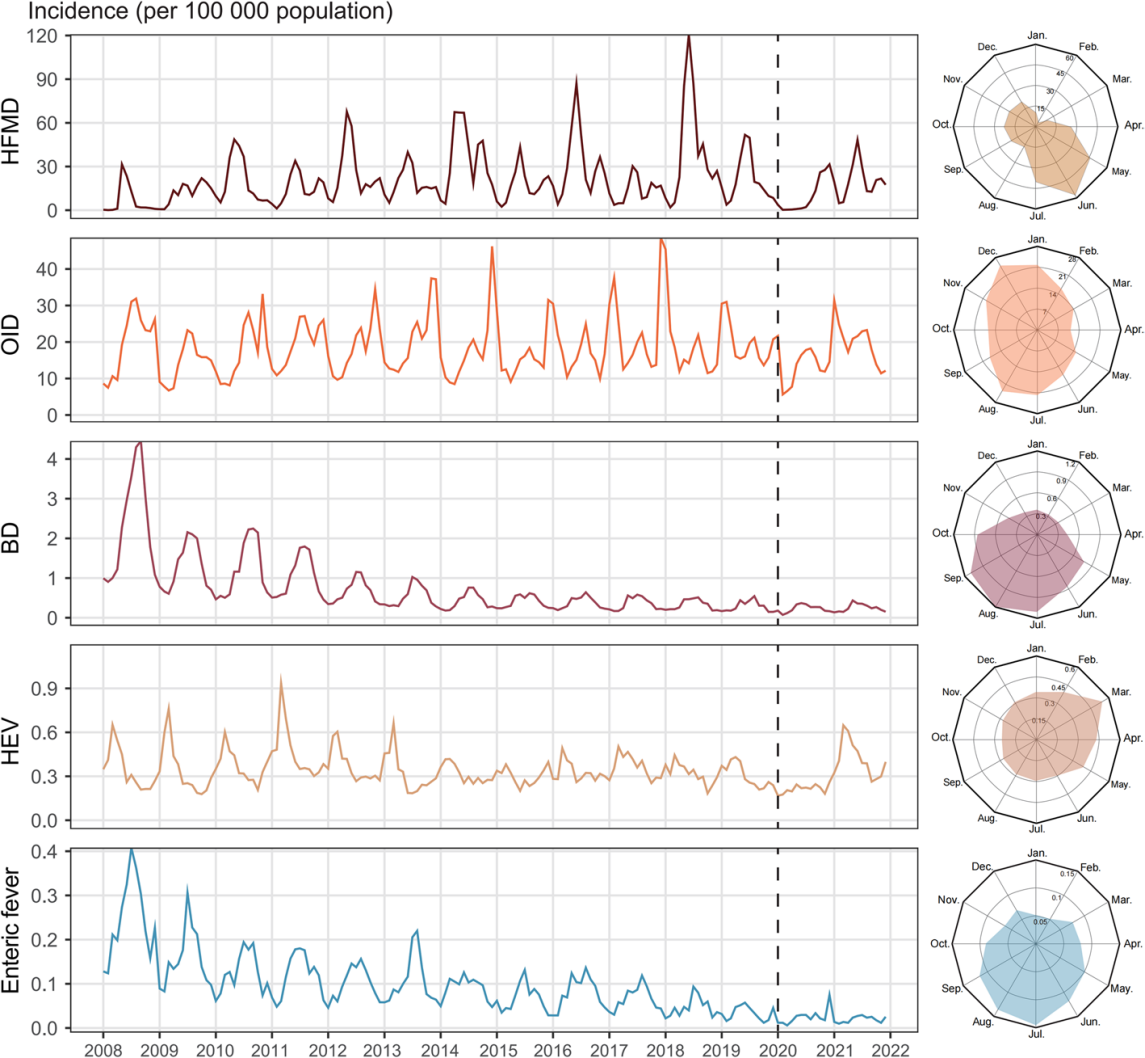
Time-series graphs and radar charts presenting seasonal patterns of intestinal infectious diseases. The radius in the radar charts represented average monthly incidence from 2008‒2021. The dashed line indicated the time of the COVID-19 outbreak in January 2020 in China

During 2008 to 2021, a total of 101 cholera, 55,298 BD, 131 AD, 5297 typhoid, 2102 paratyphoid, 27,947 hepatitis E, 1,695,925 HFMD, and 1,505,797 OID cases were reported in Zhejiang Province. Figure 2 showed the spatial distribution of IID incidence. Hangzhou had an obviously higher incidence of BD (28.49/100 000) and HEV (7.90/100 000) than any other city; The top city for enteric fever (2.23/100 000) and HFMD incidence (368.21/100 000) was Ningbo, OID incidence was mainly distributed in the North Zhejiang region including Huzhou (364.00/100 000). In general, the incidence of BD, enteric fever, cholera, and amoebiasis dysentery showed downward trends respectively; HEV and OID incidence fluctuated at certain levels; HFMD increased first and then decreased (Fig. 3, Fig. S1 and Table S1).

The incidence of IID in Zhejiang Province showed seasonality. For BD and enteric fever, temporal peaking incidences were noticed in summer (July–September); The epidemic season for HFMD spanned from May to July with a peak in Jun. The incidence of HEV reached its peak in March, and OID incidence was highest in August and December (Fig. 3).

### Spatial cluster on IID

Moran's I indices have declined in recent years for all IID incidences. All global Moran's I indices were larger than 0, which indicated significant clustering of IID incidence throughout the whole of Zhejiang every two years. The Moran's I indices for the incidence of BD were larger compared with other IID, ranging from 0.356 to 0.788, indicating more pronounced spatial clustering (Table 1).

**Table 1:** The results of the global spatial auto-correlation test on IID incidences in Zhejiang Province, 2008–2021

Time period	BD		Enteric fever		OID		HFMD		HEV	
	**Moran's I**	***P*-value**	**Moran's I**	***P*-value**	**Moran's I**	***P*-value**	**Moran's I**	***P*-value**	**Moran's I**	***P*-value**
2008–09	0.687	0.002	0.230	0.004	0.599	0.002	0.344	0.001	0.375	0.002
2010–11	0.618	0.001	0.331	0.001	0.315	0.002	0.463	0.001	0.507	0.001
2012–13	0.772	0.001	0.361	0.002	0.257	0.001	0.406	0.001	0.415	0.001
2014–15	0.788	0.002	0.365	0.001	0.323	0.002	0.615	0.001	0.538	< 0.001
2016–17	0.744	0.001	0.338	0.001	0.376	0.002	0.469	0.001	0.578	0.002
2018–19	0.520	0.002	0.325	0.001	0.280	0.001	0.325	0.001	0.176	0.001
2020–21	0.356	0.004	0.191	0.014	0.169	0.022	0.267	0.001	0.080	0.068

*BD* Bacterial dysentery, *HEV* Hepatitis E, *HFMD* Hand, foot and mouth disease

**Fig. 4:**
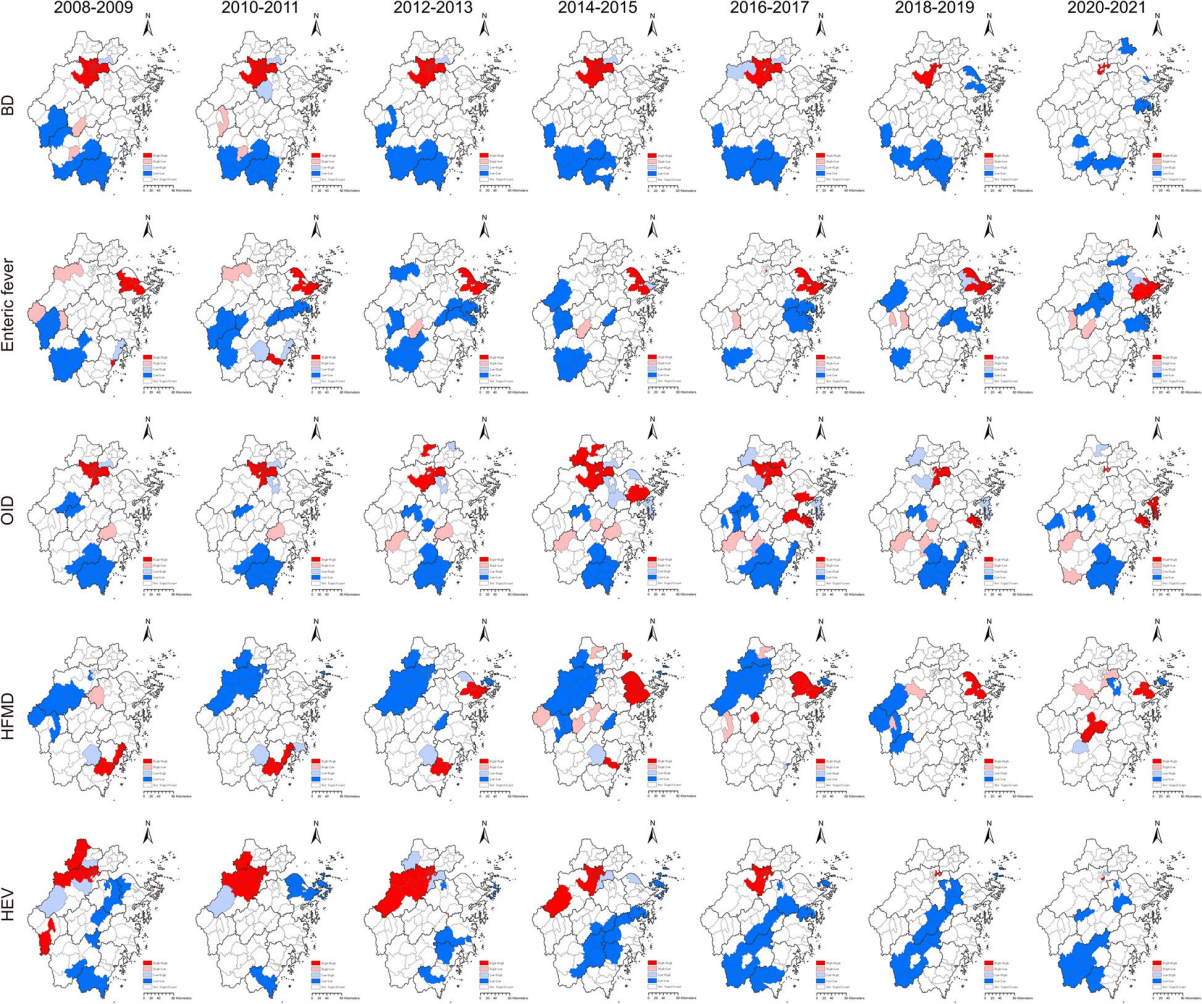
Hot spots of intestinal infectious disease incidence at the county level from 2008 to 2021 in Zhejiang Province

From 2008 to 2017, the hot spots of BD incidence were located in the urban area of Hangzhou and disappeared gradually after 2018. Cold spots of BD incidence were detected mainly in the south of Zhejiang provinces. For enteric fever, the high-high cluster was witnessed mainly in Ningbo throughout the 14 years, and the cold spots in southwest Zhejiang decreased after 2016. The hotspot regions of OID incidence in Hangzhou persisted until 2019 and new hot spots emerged in Ningbo and Taizhou in 2014. For HFMD, the hot spots in Wenzhou gradually disappeared from 2008 to 2015, while the emerging hot spots began to spread in Ningbo after 2012, followed by hot spots that emerged in Jinhua in 2020. The cold spots of HFMD incidence were mainly located in Hangzhou but gradually disappeared and were replaced by several scattered High-Low cluster regions after 2018. The High-High cluster of HEV incidence identified in Hangzhou gradually spread from 2008 to 2013 and then regressed progressively (Fig. 4 and Fig. S3).

### Associate Factors on IID

Differences in socioeconomic conditions and meteorology characteristics were evident across regions (Fig. S1 and Table S2). Multicollinearity among the covariates was not observed according to the variance inflation factors, so all variables were included in the models (Table S3). Models III containing spatial terms, a temporal term and a spatio-temporal interaction term were selected as the preferred models due to the lowest DIC (Table S4). Bayesian spatio-temporal posterior estimates of socioeconomic and climate determinants were showed in Table 2 and Table S5. Since we have standardized the covariates before fitting the models, the interpretations of RRs will be expressed based on a one-standard deviation increase in the standardized covariates.

**Table 2:** Socioeconomic and climate factors associated with each IID risk in Zhejiang Province, 2008–2021

**Covariates**	**RR (95% CI)**^a^			
	**BD**	**Enteric fever**	**HFMD**	**HEV**
Male (%)	**0.95 (0.92, 0.98)**	**0.87 (0.81, 0.93)**	0.97 (0.93, 1.01)	1.01 (0.96, 1.07)
Age < 18 year (%)	**1.37 (1.26, 1.37)**	**1.16 (1.08, 1.24)**	**1.06 (1.02, 1.10)**	**1.16 (1.12, 1.20)**
Urbanization rate (%)	1.05 (0.99, 1.11)	1.05 (0.97, 1.13)	**0.93 (0.89, 0.96)**	**1.06 (1.01, 1.10)**
GDP per capita (10 000 yuan)	**1.14 (1.10, 1.18)**	**1.18 (1.10, 1.27)**	1.02 (0.99, 1.05)	0.99 (0.96, 1.02)
Population density (1 000/ km2)	**1.29 (1.23, 1.36)**	**1.12 (1.00, 1.25)**	0.96 (0.91, 1.02)	**1.15 (1.09, 1.21)**
Aquatic product output per capita (kg)	**1.08 (1.00, 1.16)**	**0.83 (0.70, 0.95)**	0.97 (0.93, 1.02)	0.99 (0.93, 1.05)
Temperature (℃)	**1.32 (1.23, 1.41)**	**1.41 (1.33, 1.50)**	**1.22 (1.08, 1.38)**	**0.83 (0.78, 0.89)**
Surface pressure (hPa)	**0.70 (0.59, 0.83)**	1.17 (0.98, 1.39)	0.93 (0.84, 1.03)	0.98 (0.88, 1.09)
Sunlight duration (%)	1.00 (0.95, 1.04)	1.04 (0.98, 1.09)	1.03 (0.98, 1.09)	**1.12 (1.07, 1.18)**
Precipitation (cm)	0.98 (0.96, 1.01)	**1.04 (1.00, 1.08)**	**1.03 (1.00, 1.06)**	**1.05 (1.03, 1.08)**
Relative humidity (%)	**1.03 (1.00, 1.06)**	1.00 (0.96, 1.04)	**1.20 (1.16, 1.24)**	1.00 (0.97, 1.03)
Wind velocity (m/s)	0.98 (0.92, 1.04)	1.07 (0.98, 1.17)	**0.88 (0.84, 0.92)**	0.95 (0.91, 1.01)

*BD* Bacterial dysentery, *HEV* Hepatitis E, *HFMD* Hand, foot and mouth disease

^a^The interpretations of relative risks with 95% confidence intervals were expressed based on a one-standard-deviation increase in the standardized covariates. Bold indicates statistical significance

Areas with a high proportion of males had a lower risk of BD and enteric fever. People under the age of 18 may have a higher risk of IID. In socioeconomic variables, high urbanization rate was a protective factor against HFMD (RR=0.91, 95% CI: 0.88-0.94), but was a risk factor for HEV (RR=1.06, 95% CI: 1.01-1.10). BD risk (RR=1.14, 95% CI: 1.10-1.18) and enteric fever risk (RR=1.18, 95% CI: 1.10-1.27) seemed higher in areas with high GDP per capita. The greater the population density, the higher the risk of BD (RR=1.29, 95% CI: 1.23-1.36), enteric fever (RR=1.12, 95% CI: 1.00-1.25), and HEV (RR=1.15, 95% CI: 1.09-1.21). Aquatic product output per capita seemed to be positively associated with the risk of BD (RR=1.08, 95% CI: 1.00-1.16). Among climate variables, higher temperature was associated with a higher risk of BD (RR=1.32, 95% CI: 1.23-1.41), enteric fever (RR=1.41, 95% CI: 1.33-1.50), and HFMD (RR=1.22, 95% CI: 1.08-1.38), and with lower risk of HEV (RR= 0.83, 95% CI: 0.78-0.89). Precipitation was positively correlated with enteric fever (RR=1.04, 95% CI: 1.00-1.08), HFMD (RR=1.03, 95% CI: 1.00-1.06), and HEV (RR=1.05, 95% CI: 1.03-1.08). Higher HFMD risk was also associated with increasing relative humidity (RR=1.20, 95% CI: 1.16-1.24) and lower wind velocity (RR= 0.88, 95% CI: 0.84-0.92) (Table 2).

**Fig. 5:**
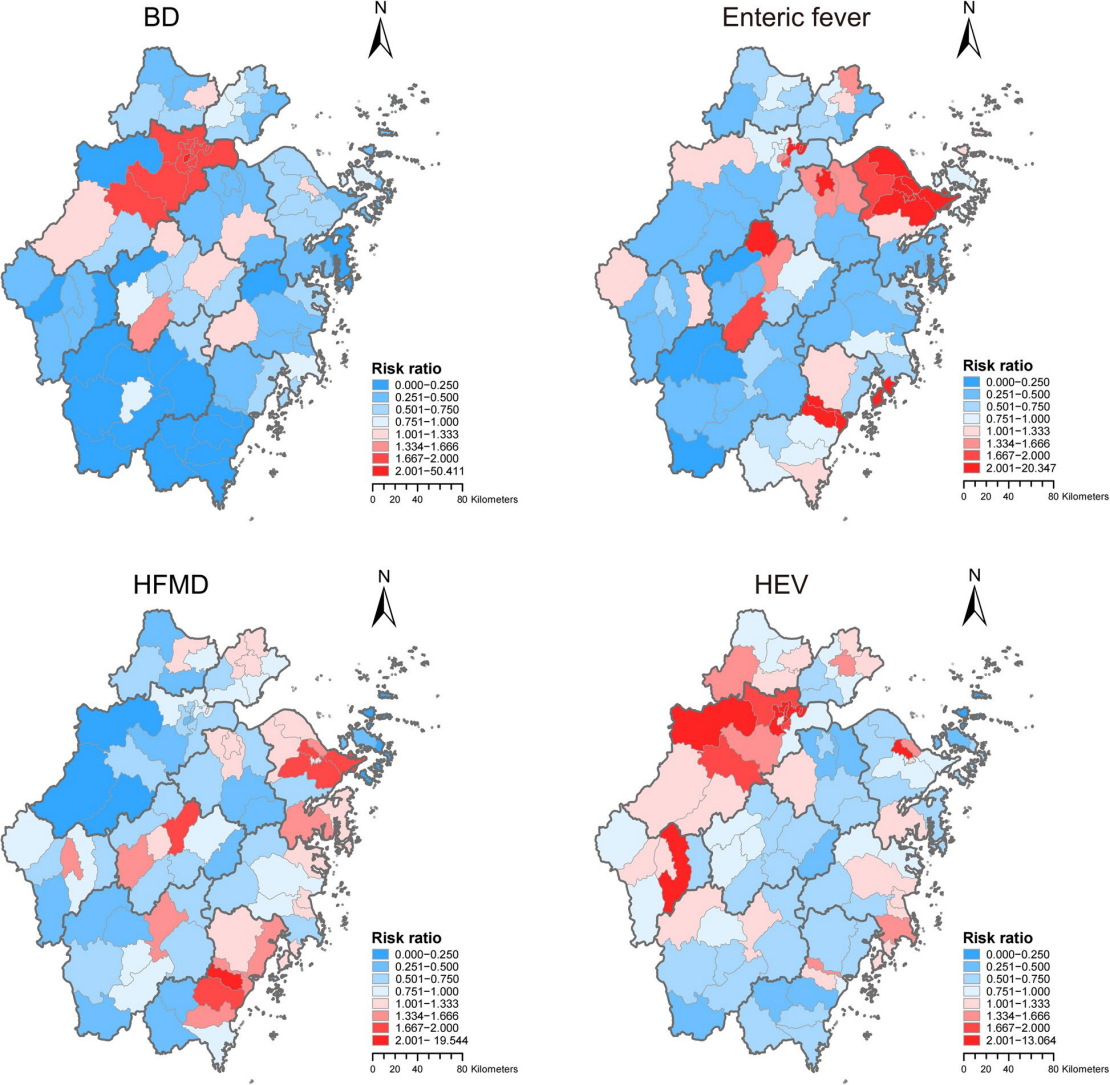
Spatial distribution of posterior means of risk ratio of the intestinal infectious diseases in Zhejiang Province, 2008–2021 based on Bayesian spatio-temporal models

The maps showing the relative risk of IID suggested higher posterior means of enteric fever risks in Ningbo, Shaoxing, and Wenzhou. BD and hepatitis E risks were higher mainly in Hangzhou. And high-risk areas for HFMD were mainly located in Ningbo, Wenzhou, and Jinhua (Fig. 5).

## Discussion

In this study of fourteen-year longitudinal surveillance in Zhejiang Province, China, we explored the secular trends, seasonality, spatial clustering distribution, and spatio-temporal epidemiological patterns in terms of socioeconomic and meteorological factors of IID.

China has made remarkable progress in enteric infectious disease control in the past few decades [7, 31]. In Zhejiang Province, thanks to the great improvement in sanitation, typhoid, paratyphoid, and dysentery presented a striking decreasing trend, but the incidence of other gastrointestinal and enterovirus diseases remained constant from 2008 to 2021. The incidence of HFMD began to decrease after 2017–2018 due to the good protective effect of enterovirus A71 vaccination introduced in 2016 [32]. Overall, the incidence of IID in each city of Zhejiang Province was significantly lower in 2020 compared with that before and rebounded in 2021. This is mainly because early COVID-19 pandemic phases with more stringent nationwide nonpharmaceutical interventions (NPI) were associated with greater reductions in IID incidence, but IID incidence rebounded in 2021 as the NPIs were relaxed [6, 33].

IID such as BD, enteric fever, OID, and HEV shared a similar transmission route and were primarily transmitted through the fecal–oral route upon ingestion of contaminated water or food [10], while HFMD was predominantly spread through direct contact [34]. Variations in the spatio-temporal epidemiological characteristics of different types of IID were observed. High incidences of BD, OID, and HEV were predominantly clustered in Hangzhou, while enteric fever and HFMD were mainly concentrated in Ningbo. This finding seems contradictory to common knowledge, as Hangzhou and Ningbo, being areas with better socioeconomic development, would be expected to be less susceptible to gastrointestinal infectious diseases. The main reason for this phenomenon can be attributed to the higher population density in these regions, which creates conditions conducive to the rapid transmission of infectious agents, leading to increased risks of gastrointestinal infectious disease outbreaks. For instance, gastrointestinal infectious diseases often tend to occur in densely populated areas like schools [35, 36]. The results of Bayesian modeling analysis indicated a positive correlation between population density and the risk of BD, enteric fever, and HEV. Another potential contributing factor could be dietary habits. Studies conducted in the United Arab Emirates and Barbados suggested this observed phenomenon might be due to the fact that higher-income households are more likely to consume food outside the home, such as fast food and junk food [37, 38]. This behavior exposes individuals to a variety of potential risks of IID, including contaminated food, improper food handling practices, and cross-contamination during food preparation and delivery. Gender and age may also be associated with IID. In Zhejiang Province, areas with a high proportion of males had a lower risk of BD and enteric fever, and people under the age of 18 may have a higher risk of IID.

The study also shed light on the impact of meteorological factors on IID risks. The incidence of IID in Zhejiang Province showed obvious seasonality, which was mainly caused by changing climatic conditions with seasons [13, 39]. Temperature as one of the most prominent roles influences the spread of infectious diseases by affecting the growth and reproduction of pathogens [40]. In Zhejiang, BD, enteric fever, and HFMD mostly occurred in the summer months and were associated with higher temperatures, which was consistent with previous studies [15–17, 41]. The temperature was negatively correlated with the risk of HEV possibly owing to that the epidemics of hepatitis E frequently clustered from February to March, during which time falls the Spring Festival in China and human activities surge [14], and the infectivity of HEV seemed to be longer at lower temperatures [42]. Another meteorological factor highly relevant to IID was strong precipitation, which was found to increase the risk of enteric fever, HFMD, and HEV. Coastal regions in Zhejiang Province were affected by typhoons, heavy rains, and floods due to tropical cyclones several times annually [43]. Studies have shown that heavy rains and floods contributed to water source pollution and pathogen transmission, resulting in a statistically significantly higher incidence of typhoid and HEV [44, 45], especially in Asian countries [46]. Wind speed was positively associated with HFMD incidence, which was in line with the results of other studies [17, 47]. One study found that higher wind speeds significantly reduced the number of infectious airborne particles in the environment, thereby diminishing the exposure to infectious particles [48]. In addition, our study found a positive association between relative humility and HFMD, which was consistent with a previous time-series study conducted in Zhejiang [49].

Since OID included several pathogens with different etiological characteristics, a modeling analysis of influencing factors on it was not performed, but its epidemiological characteristics were still worth discussing. OID was defined as infectious diarrhea other than cholera, dysentery, or typhoid/paratyphoid fever, and most prominently included rotavirus, norovirus, adenovirus, and diarrhagic *Escherichia coli* [11]. OID incidence in Zhejiang Province exhibited a seasonal characteristic and there were two peaks of OID incidence in August and December, which was largely consistent with the findings based on nationwide data [18]. This phenomenon could still be attributed to changes in meteorological factors to some extent. A meta-analysis revealed that temperature was inversely associated with enterovirus infections such as rotaviral enteritis and noroviral enteritis [50]. In humid subtropical regions with positive precipitation anomalies, heavy precipitation events were associated with an increased risk of infectious diarrhea in children [51].

The current study was known as the first comprehensive presentation of county-level research on the incidence of IID across Zhejiang Province in China, over an extended period from 2008 to 2021 when a substantial change in society, economy, environment, and health occurred. Bayesian model with spatial, temporal, and spatio-temporal interaction terms was modeled to closely investigate the spatio-temporal heterogeneities for each IID incidence at county-level, to obtain robust and comparable posterior estimates together with uncertainties for small areas. The validity of our findings has been strengthened due to the adjustment made by various meteorological and socioeconomic variables and the inclusion of spatio-temporal effects.

However, the limitations of the present study should not be ignored. Firstly, the passive surveillance data may be subjected to under-reporting as sub-clinical IID cases were unlikely to seek medical attention and be picked up by the surveillance system. Besides, the effects of exposure of climate and socioeconomic factors on IID were measured on a macroscale such as social characteristics like disparities in wealth in different counties in Zhejiang Province, rather than on the scale of individual persons, which thus leads to ecological fallacy and cannot be strong evidence for causal inference. Finally, some confounding variables such as dietary habits, quality of water source, sewage disposal and sanitation were not included.

## Conclusions

In this analysis of the spatio-temporal pattern of notifiable IID in Zhejiang Province in China from 2008 to 2021, significant between-county heterogeneity was observed. The hot spots for BD, OID, and HEV incidence were mainly in Hangzhou, while high-high cluster regions for incidence of enteric fever and HFMD were mainly located in Ningbo. Differential distributions across regions of incidence risk of enteric fever, BD, and HEV at the county level were largely explained by socioeconomic status and meteorological conditions. High-risk areas identified in this study are suggested to be prioritized for the allocation of resources for health supervision and close monitoring.

### Supplementary Information

**Additional file 1: Table S1. **Changes in the number of cases, and incidence (per 100 000) for enteric infectious diseases in Zhejiang Province, 2008**-**2021. **Fig. S1. **The number of intestinal infectious disease cases and incidence rate in Zhejiang Province from 2008 to 2021. **Table S2. **City-level climate, demographic and socioeconomic characteristics in Zhejiang Province, 2008**-**2021*. **Fig. S2. **Spatial distribution of the annual mean value of county-level socioeconomic indicators in Zhejiang Provinces in China, 2008-2021. **Fig. S3. **Annual incidence hotspots of intestinal infectious diseases at the county level in Zhejiang Province from 2008 to 2011. **Table S3.** Multicollinearity diagnosis of independent variables. **Table S4.** Model performance and selection. **Table S5**. Posterior distribution of parameters estimated by Model III for each IID incidence at the county-level in Zhejiang Province, 2008-2021. 

## Data Availability

The datasets are available from the corresponding author on reasonable request.
