# Supplementary material for: Spatio-temporal pattern and associate factors of intestinal infectious diseases in Zhejiang Province, China, 2008–2021: a Bayesian modeling study
Source: BMC Public Health. 2023 Aug 29;23:1652. doi: 10.1186/s12889-023-16552-4 (PMC10464402; doi:10.1186/s12889-023-16552-4)
Supplement: Supplementary file 1 — Additional file 1: Table S1. Changes in the number of cases, and incidence (per 100 000) for enteric infectious diseases in Zhejiang Province, 2008-2021. Fig. S1. The number of intestinal infectious disease cases and incidence rate in Zhejiang Province from 2008 to 2021. Table S2. City-level climate, demographic and socioeconomic characteristics in Zhejiang Province, 2008-2021*. Fig. S2. Spatial distribution of the annual mean value of county-level socioeconomic indicators in Zhejiang Provinces in China, 2008-2021. Fig. S3. Annual incidence hotspots of intestinal infectious diseases at the county level in Zhejiang Province from 2008 to 2011. Table S3. Multicollinearity diagnosis of independent variables. Table S4. Model performance and selection. Table S5. Posterior distribution of parameters estimated by Model III for each IID incidence at the county-level in Zhejiang Province, 2008-2021. [file 12889_2023_16552_MOESM1_ESM.docx]

**Supplementary material**

| **Table S1.** Changes in the number of cases, and incidence (per 100 000) for enteric infectious diseases in Zhejiang Province, 2008-2021 | | | | | | | | | |
| --- | --- | --- | --- | --- | --- | --- | --- | --- | --- |
| Disease | 2008 | |  | 2021 | |  | AAPC (%) for incidence, 2008-2021 |  | P-value* |
|  | No. of cases | Incidence |  | No. of cases | Incidence |  |  |  |  |
| All IID | 164674 | 351.620 |  | 247735 | 491.200 |  | 1.3 (-2.5 to 5.2) |  | 0.480 |
| Enteric fever | 1374 | 2.935 |  | 113 | 0.224 |  | -15.3 ( -17.7 to -12.8) |  | < 0.001 |
| Typhoid | 831 | 1.775 |  | 73 | 0.145 |  | -15.8 (-18.9 to -12.6) |  | < 0.001 |
| Paratyphoid | 543 | 1.160 |  | 40 | 0.079 |  | -14.2 (-17.6 to -10.8) |  | < 0.001 |
| Bacterial dysentery | 12871 | 27.493 |  | 1414 | 2.804 |  | -15.1 (-17.6 to -12.5) |  | < 0.001 |
| Hepatitis E | 1931 | 4.125 |  | 2481 | 4.919 |  | -1.8 (-4.2 to 0.8) |  | 0.153 |
| Hand, foot, and mouth disease | 36706 | 78.404 |  | 124456 | 246.770 |  | 3.9 (-3.3 to 11.7) |  | 0.268 |
| Other infectious diarrhea | 111733 | 238.663 |  | 119268 | 236.483 |  | 0.1 (-1.9 to 2.3) |  | 0.881 |
| Cholera† | 19 | 0.041 |  | 0 | 0 |  | -27.1 (-38.2 to -14.0) |  | 0.001 |
| Amoebiasis dysentery | 40 | 0.085 |  | 3 | 0.006 |  | -15.6 (-22 to -8.7) |  | 0.001 |
| AAPC: average annual percentage change. IID: intestinal infectious disease. *Joinpoint program provides significant values as *P*<0.05. †A value of zero was substituted with 1% of the smallest incidence | | | | | | | | | |


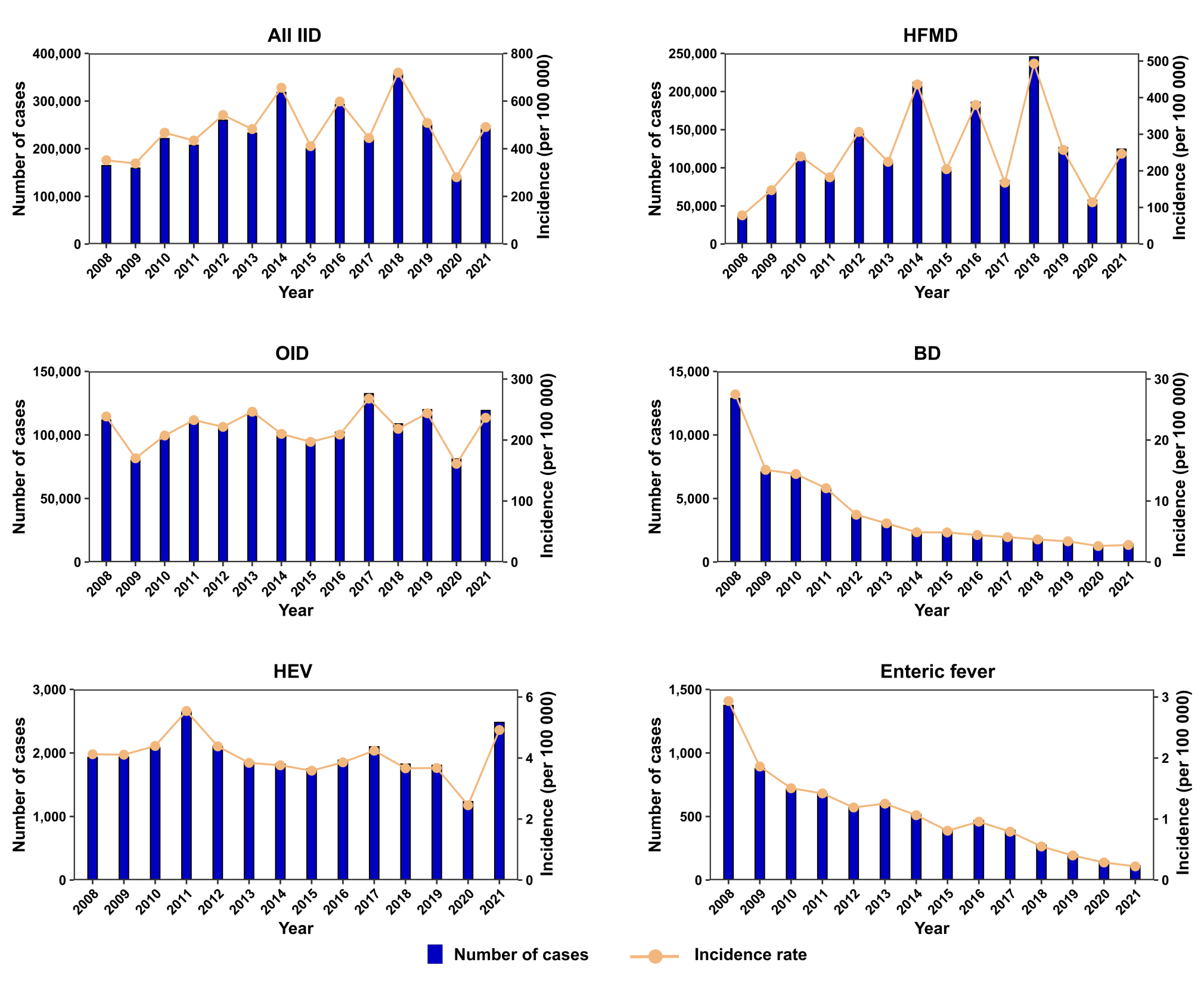


**Fig. S1 The number of intestinal infectious disease cases and incidence rate in Zhejiang Province from 2008 to 2021**

| **Table S2.** City-level climate, demographic and socioeconomic characteristics in Zhejiang Province, 2008 - 2021. * | | | | | | | | | | | |  |
| --- | --- | --- | --- | --- | --- | --- | --- | --- | --- | --- | --- | --- |
| Characteristic | Hangzhou (n=15) | Huzhou (n=5) | Jiaxing (n=7) | Jinhua (n=9) | Lishui (n=9) | Ningbo (n=11) | Quzhou (n=6) | Shaoxing (n=6) | Taizhou (n=9) | Wenzhou (n=11) | Zhoushan (n=4) | |
| Male (%) | 50.05 (49.31-50.79) | 49.4 (48.98-49.82) | 49.17 (48.84-49.5) | 51 (49.78-52.22) | 51.72 (51.37-52.07) | 49.19 (48.19-50.19) | 51.49 (50.81-52.17) | 49.91 (48.21-51.61) | 50.7 (49.07-52.33) | 51.99 (50.91-53.07) | 49.52 (49.18-49.86) | |
| Age<18 year (%) | 16.49 (13.89-19.09) | 14.91 (13.64-16.18) | 14.9 (13.4-16.4) | 17.4 (15.59-19.21) | 19.05 (17.57-20.53) | 14.92 (13.56-16.28) | 17.73 (16.42-19.04) | 15.55 (14.51-16.59) | 18.98 (17.5-20.46) | 19.73 (17.8-21.66) | 10.68 (8.81-12.55) | |
| Urbanization rate (%) | 89 (49-100) | 35 (27-52) | 50 (40-61) | 34 (18-45) | 30 (17-52) | 62 (32-76) | 22 (14-38) | 40 (30-54) | 32 (17-52) | 24 (14-45) | 44 (38-52) | |
| GDP per capita  (10 000 yuan) | 10.4 (7.7-14.8) | 7.5 (5.6-9.6) | 9.0 (6.9-10.0) | 5.6 (4.1-7.3) | 4.0 (2.6-5.8) | 10.8 (7.9-14.3) | 4.7 (2.8-6.2) | 8.7 (6.5-10.8) | 6.2 (3.7-7.9) | 4.8 (3.0-8.8) | 9.4 (7.3-11.4) | |
| Population density  (1 000/ km2) | 2.12 (0.24-5.06) | 0.47 (0.44-0.70) | 0.90 (0.76-0.98) | 0.47 (0.43-0.51) | 0.14 (0.09-0.23) | 0.77 (0.40-0.93) | 0.31 (0.23-0.72) | 0.55 (0.41-0.66) | 0.61 (0.42-1.46) | 0.92 (0.37-1.07) | 0.69 (0.64-0.74) | |
| Aquatic product output per capita (kg) | 33 (22-37) | 120 (61-183) | 54 (33-67) | 13 (9-22) | 8 (6-11) | 34 (22-244) | 20 (16-27) | 18 (7-36) | 206 (100-487) | 56 (3-98) | 1847 (1236-2860) | |
| Temperature (℃) | 18 (9-25) | 18 (10-24) | 18 (10-25) | 19 (11-25) | 18 (11-24) | 18 (10-25) | 19 (11-25) | 18 (10-25) | 18 (11-25) | 18 (12-25) | 18 (10-24) | |
| Surface pressure (hPa) | 1005 (989-1016) | 1008 (999-1017) | 1017 (1008-1023) | 983 (975-993) | 947 (933-959) | 1005 (997-1015) | 979 (972-985) | 997 (986-1006) | 999 (977-1010) | 995 (971-1006) | 1011 (1004-1017) | |
| Sunlight duration (%) | 125 (96-152) | 119 (91-145) | 127 (95-153) | 115 (90-141) | 115 (91-138) | 122 (94-151) | 117 (91-141) | 116 (92-146) | 117 (92-142) | 121 (97-147) | 135 (97-161) | |
| Precipitation (cm) | 12 (7-18) | 11 (6-17) | 10 (6-16) | 11 (7-18) | 12 (7-20) | 12 (7-18) | 11 (7-20) | 11 (6-17) | 11 (7-19) | 13 (7-20) | 11 (7-17) | |
| Relative humidity (%) | 75 (69-79) | 74 (69-79) | 75 (70-79) | 71 (65-76) | 75 (69-79) | 77 (72-81) | 71 (66-77) | 73 (68-78) | 76 (71-80) | 79 (74-83) | 80 (73-86) | |
| Wind velocity (m/s) | 4.20 (1.99-4.87) | 2.45 (2.20-2.77) | 3.68 (3.12-5.35) | 1.93 (1.79-2.09) | 1.64 (1.53-1.77) | 3.65 (2.70-5.41) | 2.00 (1.84-2.20) | 2.30 (2.10-2.65) | 2.50 (2.01-3.50) | 2.75 (1.75-4.04) | 6.43 (5.91-6.90) | |
| *All variables were expressed as median (IQR). n= number of included counties. | | | | | | | | | | | |  |


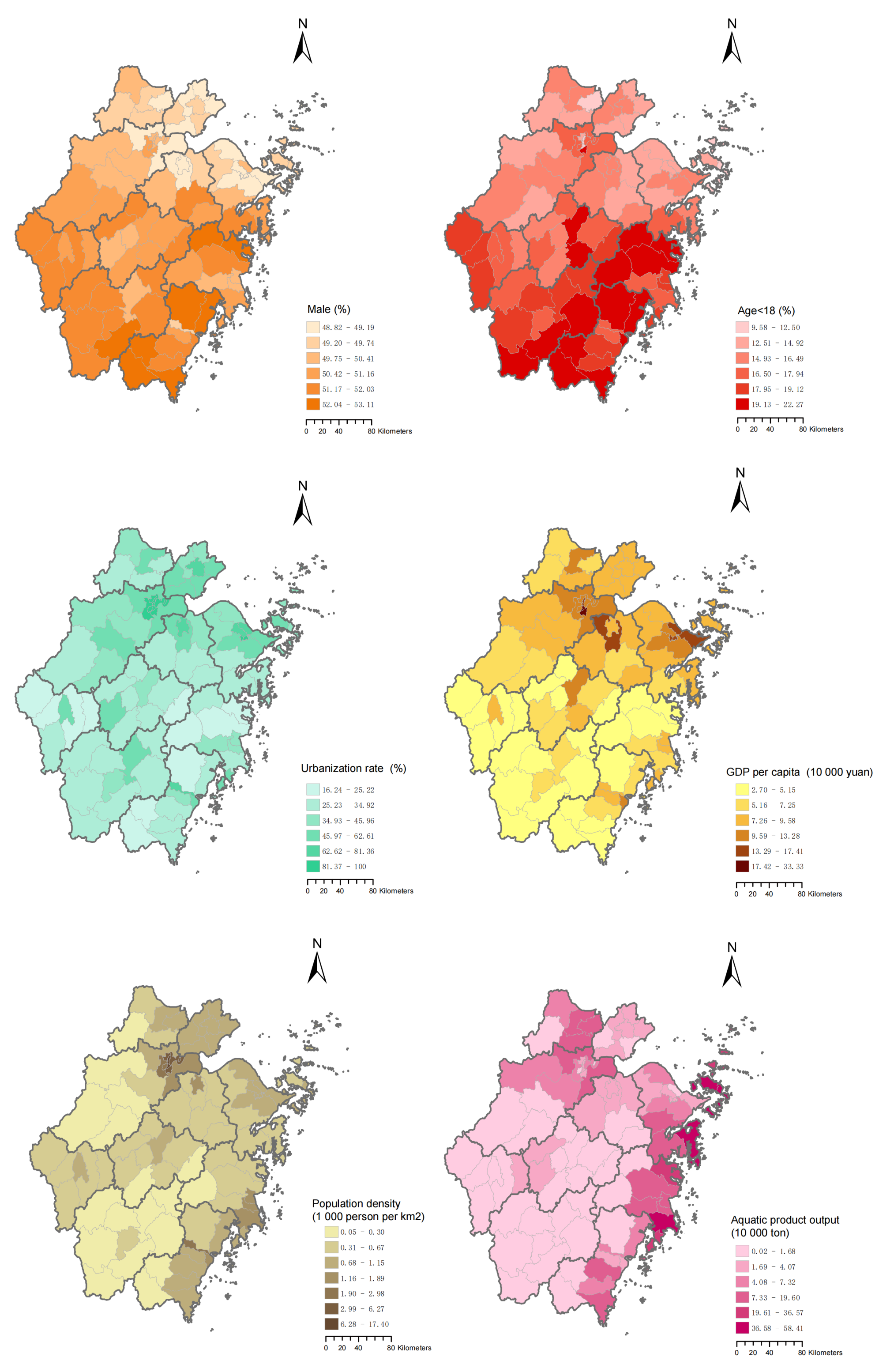


**Fig. S2 Spatial distribution of the annual mean value of county-level socioeconomic indicators in Zhejiang Provinces in China, 2008-2021.**


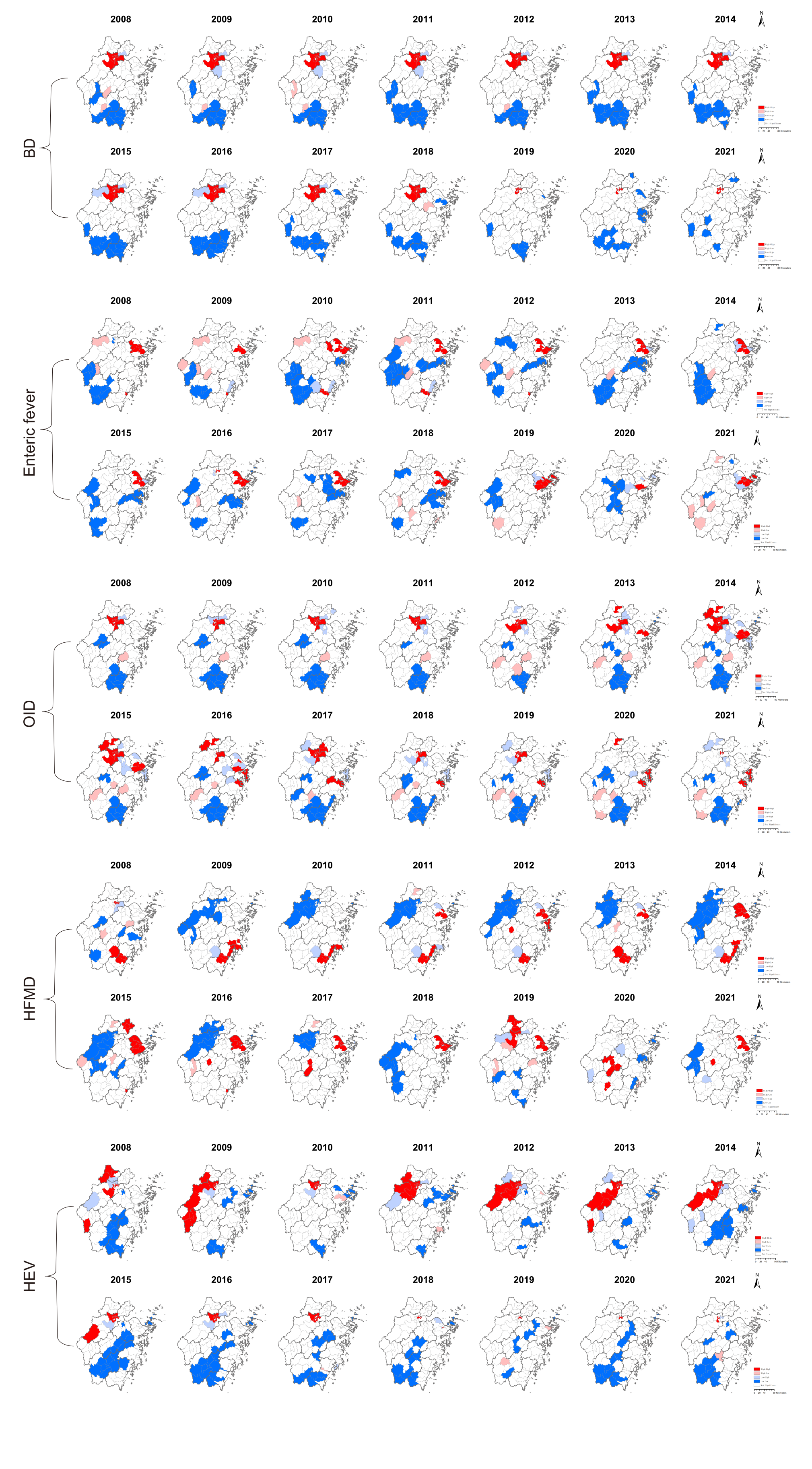


**Fig. S3 Annual incidence hotspots of intestinal infectious diseases at the county level in Zhejiang Province from 2008 to 2011.**

| **Table S3.** Multicollinearity diagnosis of independent variables. | |
| --- | --- |
| Variables | VIF |
| Age<18 year (%) | 1.67 |
| Male (%) | 2.24 |
| Urbanization rate (%) | 2.33 |
| GDP per capita (10 000 yuan) | 2.06 |
| Population density (1 000/ km^2^) | 1.55 |
| Aquatic product output per capita (kg) | 1.43 |
| Surface pressure (hPa) | 2.61 |
| Temperature (℃) | 3.31 |
| Precipitation(cm) | 1.87 |
| Sunlight duration(h) | 3.07 |
| Relative humidity (%) | 1.97 |
| Wind velocity (m/s) | 2.80 |
| VIF: Variance inflation factor | |

| **Table S4.** Model performance and selection | | | | | | |
| --- | --- | --- | --- | --- | --- | --- |
| **Model** | **Type** | **Included terms** | **DIC *** | | | |
|  |  |  | **BD** | **Enteric fever** | **HFMD** | **HEV** |
| Model I | Spatial model | $u_{i}+v_{i}$ | 47848.77 | 22241.79 | 159929.9 | 49052.07 |
| Model II | Spatial + temporal model | $u_{i}+v_{i}+\varphi_{t}$ | 47338.13 | 22228.07 | 147986.6 | 48472.75 |
| Model III | Spatial + temporal + spatiotemporal interaction model | $u_{i}+v_{i}+\varphi_{t}+\psi_{it}$ | 46595.82 | 22054.42 | 143793.2 | 48255.33 |
| * Deviation information criteria evaluating the goodness of fit of the models for each intestinal infectious disease. BD: bacterial dysentery; HEV: hepatitis E; HFMD: hand, foot and mouth disease. | | | | | | |

| **Table S5**. Posterior distribution of parameters estimated by Model III for each IID incidence at the county-level in Zhejiang Province, 2008-2021 | | | | |
| --- | --- | --- | --- | --- |
| Parameters | Mean (95% CI) | | | |
|  | **BD** | **Enteric fever** | **HFMD** | **HEV** |
| α (intercept) | -0.83 (-0.96, -0.70) | -0.45 (-0.59, -0.31) | -0.74 (-0.86, -0.62) | -0.18 (-0.25, -0.11) |
| u_i (spatially structured) | 0.88 (0.47, 1.54) | 5.59 (3.06, 12.3) | 19.77 (15.07, 27.12) | 4.89 (3.01, 7.66) |
| v_i (spatially unstructured) | 2.71 (1.99, 3.42) | 3.22 (2.09, 4.53) | 3.22 (2.36, 4.67) | 9.58 (6.38, 14.52) |
| φ_t (temporally structured) | 60.14 (46.79, 72.43) | 8068 (4613, 16400) | 1.78 (1.41, 2.27) | 36.88 (26.46, 50.75) |
| ψ_it(spatiotemporal interaction) | 5.04 (4.47, 5.62) | 3.91 (2.89, 5.08) | 3.25 (2.91, 3.61) | 8.18 (6.39, 10.47) |
| BD: bacterial dysentery; HEV: hepatitis E; HFMD: hand, foot and mouth disease. | | | | |
